# Potential application of mesenchymal stromal cells as a new therapeutic approach in acute respiratory distress syndrome and pulmonary fibrosis

**DOI:** 10.1186/s12931-024-02795-1

**Published:** 2024-04-18

**Authors:** Giulia Gazzaniga, Marta Voltini, Alessandro Carletti, Elisa Lenta, Federica Meloni, Domenica Federica Briganti, Maria Antonietta Avanzini, Patrizia Comoli, Mirko Belliato

**Affiliations:** 1 SC Anestesia e Rianimazione 2, Fondazione IRCCS Policlinico San Matteo, Viale Camillo Golgi 19, Pavia, PV 27100 Italy; 2 Department of Clinical-Surgical, Diagnostic and Pediatric Sciences, University of Pavia, Pavia, Italy; 3 Cardio-Thoracic Surgery Department, Heart & Vascular Centre, Maastricht University Medical Centre (MUMC+), P. Debyelaan 25, Maastricht, 6229 HX The Netherlands; 4 SC Anestesia e Rianimazione 3 – TIPO, Fondazione IRCCS Policlinico San Matteo, Pavia, Italy; 5 SSD Cell Factory and Center for Advanced Therapies, Fondazione IRCCS Policlinico San Matteo, Pavia, Italy; 6 UOS Transplant Center, Fondazione IRCCS Policlinico San Matteo, Pavia, Italy; 7 Department of Internal Medicine, University of Pavia, Pavia, Italy; 8 Pediatric Hematology/Oncology Unit, Fondazione IRCCS Policlinico San Matteo, Pavia, Italy

**Keywords:** Acute respiratory distress syndrome, ARDS, Pulmonary fibrosis, Mesenchymal stromal cells, COVID-19

## Abstract

While the COVID-19 outbreak and its complications are still under investigation, post-inflammatory pulmonary fibrosis (PF) has already been described as a long-term sequela of acute respiratory distress syndrome (ARDS) secondary to SARS-CoV2 infection. However, therapeutical strategies for patients with ARDS and PF are still limited and do not significantly extend lifespan. So far, lung transplantation remains the only definitive treatment for end-stage PF. Over the last years, numerous preclinical and clinical studies have shown that allogeneic mesenchymal stromal cells (MSCs) might represent a promising therapeutical approach in several lung disorders, and their potential for ARDS treatment and PF prevention has been investigated during the COVID-19 pandemic. From April 2020 to April 2022, we treated six adult patients with moderate COVID-19-related ARDS in a late proliferative stage with up to two same-dose infusions of third-party allogeneic bone marrow-derived MSCs (BM-MSCs), administered intravenously 15 days apart. No major adverse events were registered. Four patients completed the treatment and reached ICU discharge, while two received only one dose of MSCs due to multiorgan dysfunction syndrome (MODS) and subsequent death. All four survivors showed improved gas exchanges (PaO2/FiO2 ratio > 200), contrary to the others. Furthermore, LDH trends after MSCs significantly differed between survivors and the deceased. Although further investigations and shared protocols are still needed, the safety of MSC therapy has been recurrently shown, and its potential in treating ARDS and preventing PF might represent a new therapeutic strategy.

## To the Editor

Pulmonary fibrosis (PF) is a relatively rare but severe condition characterized by reduced lung compliance and function. Despite having a multifactorial etiology, post-inflammatory PF can be the consequence of severe pulmonary infection and acute respiratory distress syndrome (ARDS). While the COVID-19 outbreak and its complications are still under investigation, ARDS-related PF has already been described as a long-term sequela. However, pharmacologic therapy has been largely ineffective for patients with ARDS, and management mainly focuses on supportive care measures. Likewise, PF has limited treatment options, as currently approved therapies do not significantly extend lifespan. So far, lung transplantation remains the only definitive treatment for end-stage PF, though this option is not always available and is associated with peri-operative high morbidity and mortality and poor long-term survival.

Over the last few years, numerous preclinical and clinical studies have shown that advanced therapy medicinal products (ATMP) based on allogeneic mesenchymal stromal cells (MSCs) might represent a promising therapeutical approach in several lung disorders [1–3]. Several data have described the potential of MSCs and their ability to migrate to a site of injury and guide tissue regeneration. Moreover, when intravenously administered, 50–80% of MSCs tend to localize in the lungs with a first-pass effect [4]. Furthermore, since MSCs do not express the two primary human receptors for host-pathogen interaction in SARS-CoV-2 infection [5], their potential for ARDS treatment and PF prevention has been exploited during the COVID-19 pandemic.

From April 2020 to April 2022, we treated six adult patients in mechanical ventilation for moderate COVID-19-related ARDS (median PaO_2_/FiO_2_ ratio 130, median Crs 26.5 cmH_2_O) in a late proliferative stage with third-party allogeneic bone marrow-derived MSCs (BM-MSCs) on a compassionate use basis [6]. The work was approved by the local Ethics Committee, and conducted in accordance with the Declaration of Helsinki.

The patients (1 female and 5 males) had a median age of 65 years (44–76 year) and a median body mass index (BMI) of 27.8 [(25.8–33.9) IQR 3.03] and received up to two same-dose infusions (1 × 10^6^/kg body weight) of BM-MSCs, administered intravenously 15 days apart. They were then monitored and considered for subsequent monthly BM-MSC infusions if signs of PF were observed. All subjects had already been treated with pronation cycles, myorelaxants, dexamethasone, and antibiotics according to international and national guidelines. Moreover, four patients had also received hyperimmune plasma for COVID-19 before BM-MSC’s first infusion (median 15 days). The cohort’s demographic and clinical characteristics are described in Table 1.

**Table 1:** Baseline demographic, clinical characteristics, and outcomes of the patients who developed acute respiratory distress syndrome (ARDS) SARS-CoV2-related and who have been enrolled in this study

	Survivors (*N* = 4)	Non-survivors (*N* = 2)	Total (*N* = 6)
Age, years (mean ± SD)	58.2 ± 13.9	71 ± 3.5	62.6 ± 12.9
Sex, N (%)			
Male	3	2	5 (83.33%)
Female	1	0	1 (16.77%)
Body mass index (median, range)	28.5 (26.7–33.9)	26.7 (25.8–27.7)	27.8 (25.8–33.9)
Charlson Comorbidity Index (median)	2	3.5	3
Comorbidities, N (%)			
- Tabagic habit	0 (0%)	0 (0%)	0 (0%)
- Type 2 diabetes mellitus (T2DM)	1 (25%)	0 (0%)	1 (16.66%)
- Arterial hypertension (AH)	2 (50%)	0 (0%)	2 (33.33%)
- Chronic obstructive pulmonary disease (COPD)	0 (0%)	0 (0%)	0 (0%)
- Immunosuppression	0 (0%)	0 (0%)	0 (0%)
SOFA score (median)	5.5	7	6.5
WHO ordinal scale score (median)	4	4	4
PaO_2_/FiO_2_ ratio (mean ± SD)	135 ± 36.9	89 ± 14.8	119.8 ± 37.6
Crs, cmH_2_O (mean ± SD)	28.2 ± 4.7	27.5 ± 10.6	28 ± 6
Days in ICU (mean ± SD)	58.7 ± 30.6	30.5 ± 0.7	49.3 ± 27.8
Days of mechanical ventilation (mean ± SD)	28 ± 18.1	29.5 ± 0.7	28.5 ± 14.1
Steroid treatment, N (%)	100%	100%	100%
Antibiotic treatment, N (%)	100%	100%	100%
Antiviral treatment, N (%)	0%	0%	0%
Hyperimmune plasma treatment, N (%)	75%	50%	66.66%
WBC, 10^9^/L (media ± SD)	17.0 ± 10.3	20.2 ± 10	18.7 ± 9.2
Lymphocytes, 10^9^/L (mean ± SD)	4.2 ± 5.6	0.8 ± 0.2	3.1 ± 4.7
CRP, mg/dL (mean ± SD)	6.55 ± 4.1	4.8 ± 6.7	5.9 ± 4.4
PCT, ng/mL (mean ± SD)	0.3 ± 0.2	0.3 ± 0.0	0.3 ± 0.1
LDH, U/L (mean ± SD)	670 ± 281	370 ± 117	570 ± 272

SOFA sequential organ failure assessment; PaO_2_ partial pressure (arterial) of oxygen; FiO_2_ fraction of inspired oxygen; Crs compliance of the respiratory system; ICU intensive care unit; WBC white blood cells; CRP C-reactive protein; PCT procalcitonine; LDH lactate dehydrogenase

Four patients completed the treatment, while the remaining two received only one dose of MSCs due to a rapid deterioration in their clinical conditions and exitus after the onset of septic shock and multiorgan dysfunction syndrome (MODS). However, the other four patients were successfully discharged from the Intensive Care Unit (ICU) and are still alive at 1-year follow-up. In this regard, several studies have widely discussed mortality as a primary outcome after MSC therapy, but consistent results still need to be provided. However, a decreased length of hospital stay might be a better indicator of efficacy since it entails improved clinical conditions and reduced mortality [7, 8].

**Fig. 1:**
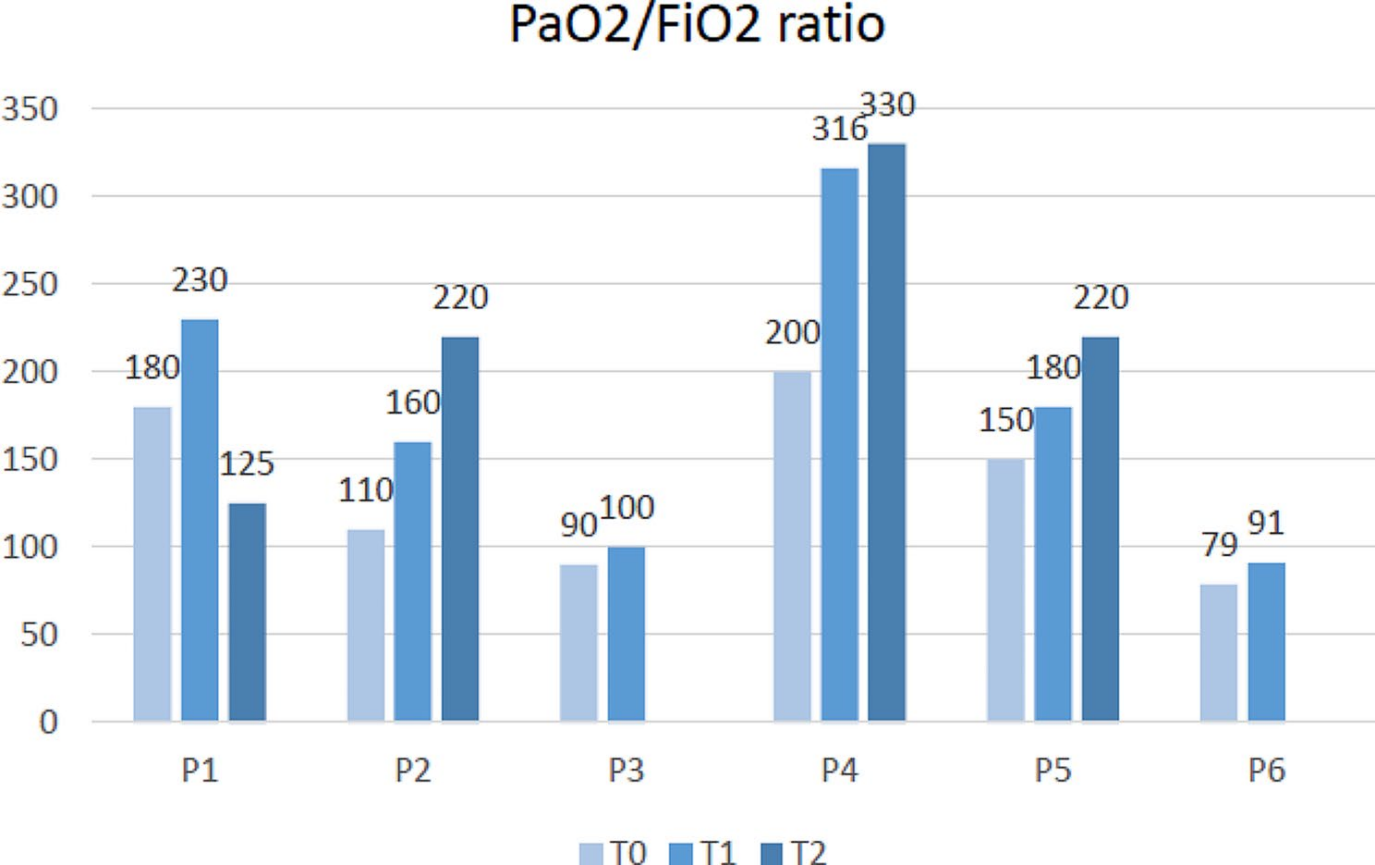
Trends of PaO_2_/FiO_2_ ratio of each patient at the time of enrollment (T0), within 24 h after the infusion of the first (T1) and second (T2) dose of BM-MSCs, administrated 15 days apart PaO_2_ partial pressure (arterial) of oxygen; FiO_2_ fraction of inspired oxygen

Regarding clinical outcomes, all patients showed improved gas exchange after the first dose of MSCs (Fig. 1), but that was insufficient to hinder the disease progression in those with severe ARDS (P3 and P6). Nevertheless, the other individuals showed an increase in PaO_2_/FiO_2_ ratio > 200 either after the first (P1 and P4) or the second (P2 and P5) MSC infusion, thus evolving in mild ARDS. However, clear radiological signs of improvement were not detected after the last administration, though survivors showed moderate resolution of bilateral parenchymal damage or lack of deterioration, as reported in the literature [9]. Still, notable lung structure changes may take some time after MSC treatment. Finally, no patients required further monthly BM-MSC infusions.

**Fig. 2:**
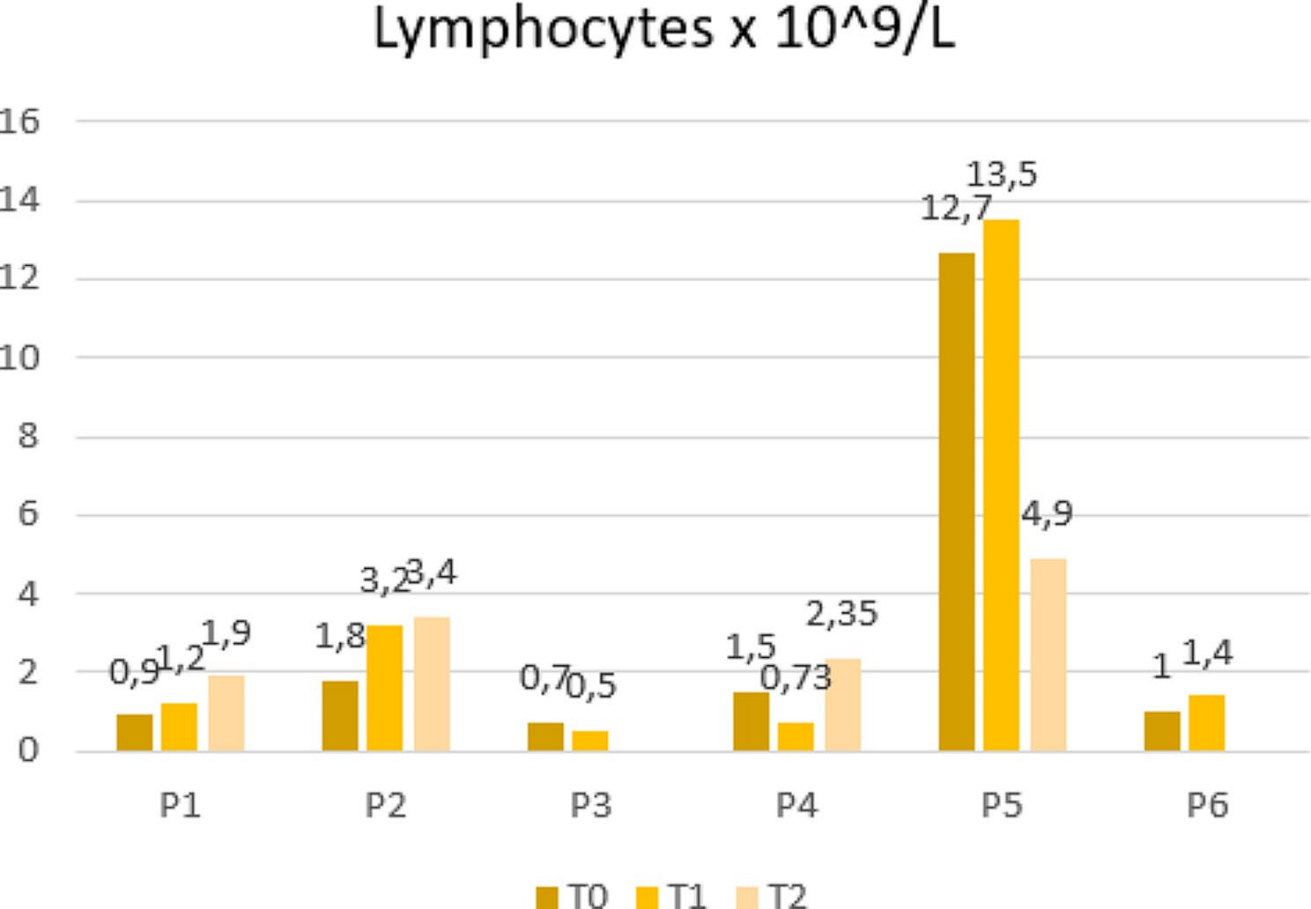
Trends of lymphocytes’ levels of each patient at the time of enrollment (T0), within 24 h after the infusion of the first (T1) and second (T2) dose of BM-MSCs, administrated 15 days apart

**Fig. 3:**
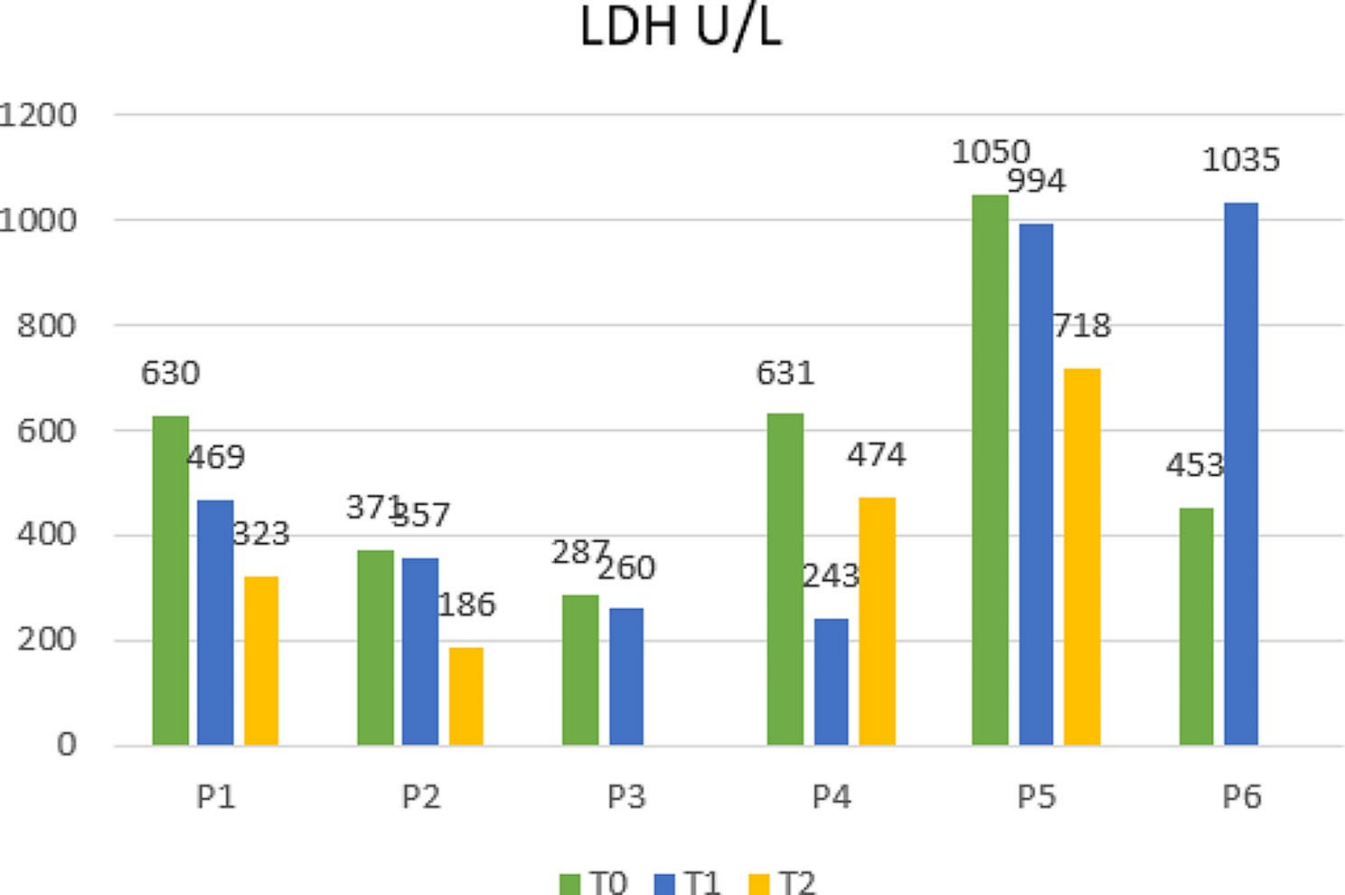
Trends of lactate dehydrogenase (LDH) levels of each patient at the time of enrollment (T0), within 24 h after the infusion of the first (T1) and second (T2) dose of BM-MSCs, administrated 15 days apart

**Fig. 4:**
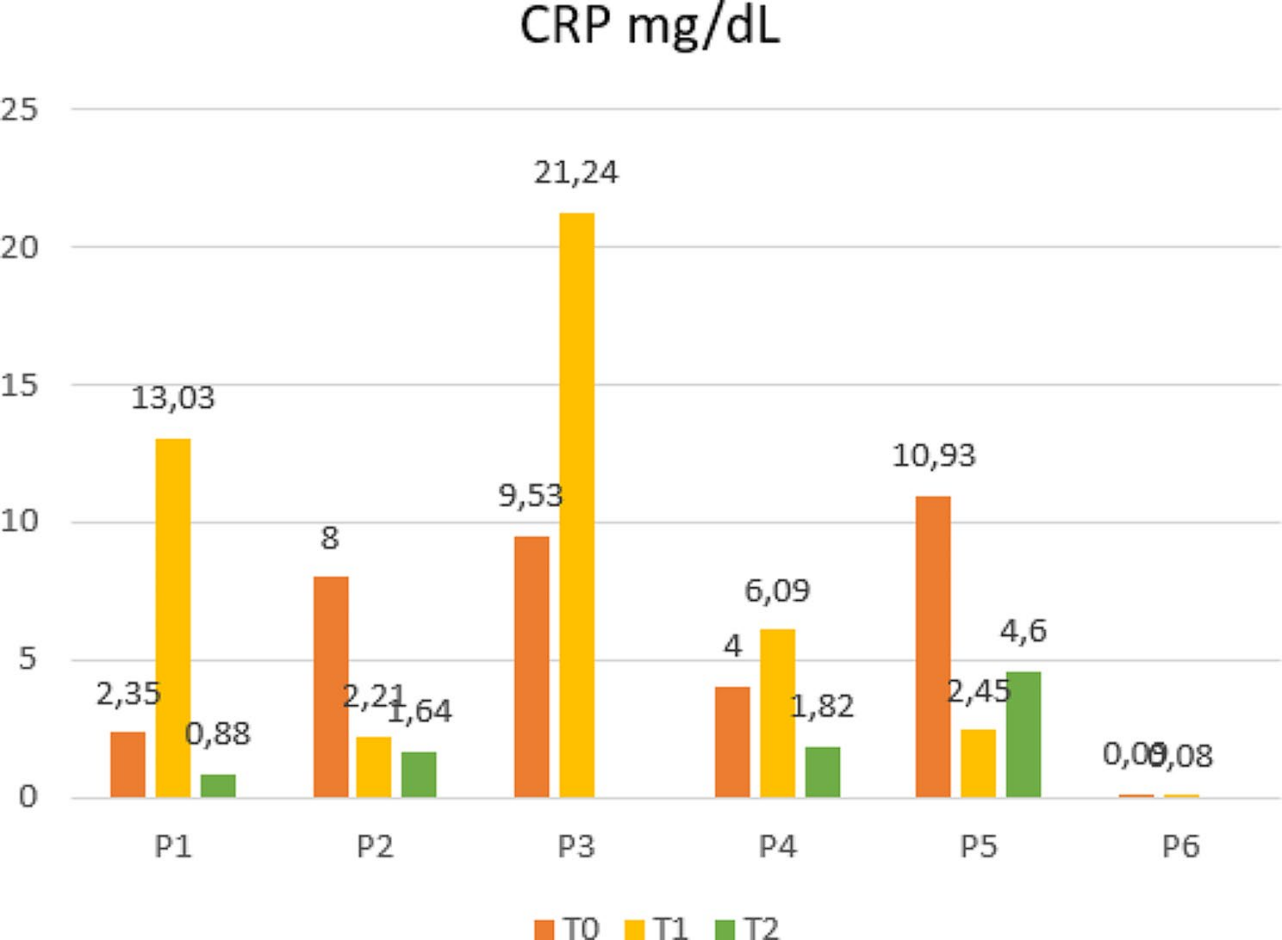
Trends of C-reactive protein (CRP) levels of each patient at the time of enrollment (T0), within 24 h after the infusion of the first (T1) and second (T2) dose of BM-MSCs, administrated 15 days apart

From the laboratory’s perspective, we observed an improvement in lymphocyte numbers in survivors, while patients who died still displayed lower levels after the last dose [10] (Fig. 2). Furthermore, lactate dehydrogenase (LDH) trends after MSCs differed between survivors and the deceased (Fig. 3). High LDH blood levels are a biomarker commonly associated with higher mortality and poor prognosis in several conditions, including ARDS [11]. Recent studies on COVID-19 patients documented a correlation between high levels of LDH and the severity of the disease and intra-hospital mortality [12]. Regarding inflammation markers, C-reactive protein (CRP) trends declined in all cases except one (P3) but did not show any substantial correlation with outcomes (Fig. 4). However, in the literature, the association of this parameter with MSC treatment is controversial since some studies reported no differences in CRP trends between cases and controls, while others documented inferior blood values in MSC recipients [13]. Moreover, some authors have suggested that steroids and hyperimmune plasma may mitigate the anti-inflammatory effect of MCSs [14]. Furthermore, despite initially not being considered subject to rejection, recent studies have indicated that MSCs may be influenced by the host’s immune response, particularly in the inflammatory milieu and hypoxia [15].

The literature has extensively documented the pleiotropic anti-inflammatory and regenerative effects of MSCs, and this ATMP likely plays a significant role in ameliorating COVID-19 through immunomodulation. However, it is essential to mention that MSCs may have modest constitutive immune-modulating properties. Some studies have described the potential discrepancies in MSC phenotype induction when exposed to the host’s pro-inflammatory mediator [16]. In patients with severe or critical COVID-19, high levels of pro-inflammatory cytokines during MSC treatment may induce a stronger phenotype, even without prior ex vivo priming, while in patients with mild or moderate COVID-19, the immune modulatory phenotype may be less effective. Moreover, age-related quantitative and qualitative changes in the immune system may affect the host’s immune response to MSC treatment [17]. Furthermore, the interaction between allogenic and resident MSCs is still under investigation [18].

Finally, no major adverse events were registered after BM-MSC administration, including venous thromboembolism (VTE) or pulmonary embolism (PE). The safety of MSC therapy in COVID-19 patients has already been documented in the literature. A recent randomized study of BM-MSCs versus placebo in early-onset COVID-19-related ARDS reported the absence of infusion-related toxicities and similar serious adverse events over 30 days between the enrolled groups [19]. Data from systematic reviews also described the lack of significant adverse effects after MSC therapy [13] or reported mild adverse events that resolved spontaneously or with minimal supportive treatment in all patients [14].

Although the results of MSC treatment in ARDS and PF are encouraging, additional information from controlled studies should be obtained regarding MSCs source, administration schedule, and dose to design a shared clinical protocol for BM-MSC therapy in early chronic lung injury and prevention of PF secondary to post-infective ARDS. Moreover, a deeper understanding of the interactions between infused third-party allogeneic MSCs and lung-resident cellular populations, including resident MSCs, macrophages, and lymphocytes, might offer valuable insight into the pathogenesis of PF and provide novel therapeutic tools [18, 20, 21]. In conclusion, the dual nature of MSCs in developing and treating post-inflammatory fibrotic diseases represents an inviting challenge for research and might have pivotal applications in clinical settings.

## Data Availability

The datasets used and analysed during the current study are available from the corresponding author on reasonable request.

## Abbreviations

ARDS: acute respiratory distress syndrome
ATMP: advanced therapy medicinal products
BMI: body mass index
BM-MSCs: bone marrow-derived MSCs
COVID-19: coronavirus disease 19
CRP: C-reactive protein
ICU: intensive care unit
LDH: lactate dehydrogenase
MSCs: mesenchymal stromal cells
MODS: multiorgan dysfunction syndrome
PF: pulmonary fibrosis
PE: pulmonary embolism
SARS-CoV-2: severe acute respiratory syndrome coronavirus 2
VTE: venous thromboembolism
